# Associação entre Escores de Risco Clínico (HEART, GRACE e TIMI) e Complexidade Angiográfica na Síndrome Coronária Aguda sem Elevação do Segmento ST

**DOI:** 10.36660/abc.20190417

**Published:** 2021-08-09

**Authors:** Alexandre Vianna Cedro, Diandro Marinho Mota, Louis Nakayama Ohe, Ari Timerman, José Ribamar Costa, Laura de Siqueira Castro

**Affiliations:** 1 Instituto Dante Pazzanese de Cardiologia São PauloSP Brasil Instituto Dante Pazzanese de Cardiologia, São Paulo, SP - Brasil; 2 Universidade Federal de São Paulo Escola Paulista de Medicina São PauloSP Brasil Universidade Federal de São Paulo Escola Paulista de Medicina, São Paulo, SP - Brasil

**Keywords:** Síndrome Coronária Aguda, Escore de Disfunção Orgânica, Hospitalização, Trombose, Infarto do Miocárdio, Angiografia/Complicações

## Abstract

**Fundamentos:**

Os escores GRACE, TIMI e HEART foram validados para avaliar desfechos clínicos desfavoráveis no contexto da suspeita de síndrome coronária aguda sem elevação do segmento ST (SCASSST). No entanto, a associação entre os escores clínicos e a complexidade angiográfica ainda não está bem estabelecida.

**Objetivos:**

Descrever as características clínicas de pacientes com SCASSST admitidos em internação hospitalar para estratificação invasiva, a fim de avaliar a associação entre os escores clínicos (TIMI, GRACE e HEART) e a complexidade angiográfica, através do escore SYNTAX.

**Métodos:**

Estudo observacional incluindo pacientes com diagnóstico de SCA e admitidos no Instituto Dante Pazzanese de Cardiologia entre os meses de julho de 2018 e fevereiro de 2019. A associação entre os escores foi avaliada por correlações bivariadas e a sua acurácia pela área sob a curva (ASC) ROC. A significância estatística foi estabelecida em 5% (p < 0,05).

**Resultados:**

Um total de 138 pacientes foram diagnosticados com SCASSST. As medianas do GRACE, TIMI e HEART foram de 97, 3 e 5, respectivamente. A mediana do SYNTAX foi de 8. Foram observadas correlações positivas do SYNTAX com o HEART (ρ = 0,29; p < 0,01) e o GRACE (ρ = 0,18; p < 0,03). Em contrapartida, a correlação com o TIMI não atingiu significância estatística (ρ = 0,15; p = 0,08). O HEART foi o escore com a maior ASC para predizer SYNTAX > 32 [HEART = 0,81] (IC 95% 0,7-0,91). HEART > 4 apresentou sensibilidade de 100%, com especificidade de 50%, e GRACE > 139 sensibilidade de 55% e especificidade de 97% para SYNTAX alto.

**Conclusão:**

Os escores clínicos de risco apresentam associação modesta ao escore SYNTAX. O uso combinado do HEART e do GRACE, entretanto, oferece acurácia favorável para a detecção de complexidade angiográfica.

## Introdução

A síndrome coronária aguda sem elevação do segmento ST (SCASSST) apresenta amplo espectro de gravidade, que varia de acordo com as características eletrocardiográficas, clínicas e laboratoriais. Dessa forma, a estratificação de risco é fundamental em todo paciente com SCASSST e tem implicação direta na conduta inicial. Está demonstrado que a utilização de modelos multivariados representa a forma mais acurada na predição de risco, e é superior à impressão clínica individual.^1^^,^^2^

Os escores Thrombolysis in Myocardial Infarction (TIMI), Global Registry for Acute Coronary Events (GRACE) e Heart Score (HEART) são os mais utilizados no cenário de dor torácica na sala de emergência e foram validados para predizer desfechos clínicos indesejados. Entretanto, essas pontuações não se destinam a estimar a extensão da doença arterial coronariana.^3^^–^^6^

Assim como a análise dos escores clínicos, a avaliação da complexidade anatômica através do escore SYNTAX é fundamental na definição da estratégia de revascularização miocárdica e apresenta implicação prognóstica notória.^7^

O estudo SYNTAX, que deu origem ao escore, comparou desfechos clínicos tardios em pacientes multiarteriais tratados com angioplastia (ICP) ou cirurgia de revascularização miocárdica (RM). Observou-se resultado favorável à RM nos pacientes com doença coronariana mais extensa (SYNTAX ≥ 33).^8^ Desse modo, a determinação do SYNTAX pode interferir também na abordagem clínica, ao auxiliar na decisão da terapia de dupla antiagregação plaquetária quando a anatomia for favorável à abordagem cirúrgica.^7^^,^^8^

Apesar da importância em identificar fatores prognósticos da extensão da doença coronariana, poucos estudos avaliaram a correlação entre os escores clínicos e a complexidade anatômica. Resultados controversos estão sendo vistos nessa relação quando utilizados os escores TIMI e GRACE, e não existem dados na literatura médica que associem a pontuação do HEART à extensão da doença arterial coronariana.^9^^–^^11^

O presente estudo tem, portanto, o objetivo de avaliar a associação entre os escores de risco TIMI, GRACE e HEART^12^ e a complexidade da doença coronariana avaliada pelo escore SYNTAX em pacientes com SCASSST submetidos à coronariografia.

## Métodos

### Seleção da população

Trata-se de um estudo observacional e longitudinal realizado no Instituto Dante Pazzanese de Cardiologia (São Paulo, Brasil) entre os meses de julho de 2018 e fevereiro de 2019, aprovado pelo Comitê de Ética em pesquisa da instituição. Todos os pacientes assinaram o termo de consentimento livre e esclarecido no momento da internação.

Foram incluídos todos os pacientes com mais de 18 anos que apresentavam diagnóstico de SCASSST no setor de emergência e que foram submetidos à coronariografia durante a internação no período da coleta de dados. Pacientes com RM, infarto agudo do miocárdio (IAM) com supradesnivelamento do segmento ST (IAMCSST) ou com bloqueio de ramo esquerdo supostamente novo foram excluídos.

### Escores clínicos

Todos os pacientes foram avaliados e estratificados com escores HEART, TIMI e GRACE no momento da internação. Para calcular os respectivos escores, foram utilizados dados clínicos, o eletrocardiograma da admissão, a primeira dosagem de creatinina e o maior valor da troponina das primeiras 12 horas de atendimento. Foi definida como troponina elevada quando o valor de troponina T ultrassensível é igual ou maior que 0,01 ug/L, ou seja, maior do que o percentil 99 da população geral.

No cálculo dos escores foram utilizados os critérios previamente definidos nos respectivos trabalhos de validação. A análise do TIMI foi realizada através das sete variáveis dicotômicas habituais. A presença de cada variável acrescentou um ponto ao escore total, que varia de zero a sete. O escore TIMI considerado elevado foi aquele com pontuação de cinco a sete.^3^

O escore GRACE foi calculado pela análise de suas oito variáveis e revisado através de sua calculadora de pontuação oficial (*http://www.grace.org*). O escore final foi considerado elevado quando maior que 139, conforme recomendado pelas principais diretrizes.^1^^,^^2^

O escore HEART foi pontuado de 0 a 10 de acordo com suas cinco variáveis convencionadas (história, ECG, idade, fatores de risco e valor de troponina). Após o cálculo, foram classificados como alto risco aqueles pacientes com pontuação entre 7 e 10.^5^

Após a realização da estratificação invasiva, todos os cateterismos foram analisados pelo mesmo cardiologista intervencionista, com auxílio da ferramenta de Análise Coronariana Quantitativa (QCA), sem acesso aos dados clínicos e sem envolvimento com a assistência aos pacientes. O escore SYNTAX foi calculado e revisado pelo uso de calculadora de pontuação oficial (http://www.syntaxscore.com), por meio de instruções e programas disponíveis na página.

Foram avaliadas artérias com diâmetro igual ou maior que 1,5 mm e estenose igual ou maior que 50%. A pontuação foi realizada para cada paciente de acordo com os seguintes parâmetros: dominância, número de lesões, presença de oclusão crônica, trifurcação, bifurcação, lesão ostial, tortuosidade grave, calcificação, trombo e comprimento da lesão maior que 20 mm. A bifurcação foi definida quando o estreitamento de 50% da luz ocorre a 3 mm da carina, em artéria com ramos de pelo menos 1,5 mm. A calcificação coronariana grave foi determinada quando a lesão radiopaca foi observada antes mesmo da injeção do contraste. Após o cálculo do escore, cada paciente foi classificado como SYNTAX baixo (igual ou menor a 22), moderado (23-32) ou alto (igual ou maior que 33).^7^

### Análise estatística

Os dados estão apresentados como frequência absoluta e com valor percentual para as variáveis categóricas e como média ± desvio-padrão (DP) ou mediana com intervalo interquartil para as variáveis contínuas, conforme critérios de normalidade e distribuição. Para comparar as proporções entre os grupos, foram utilizadas tabelas cruzadas com o teste do Qui-quadrado, para se comparar variáveis contínuas entre os grupos definidos pelo SYNTAX escore utilizou-se de Modelos Lineares Generalizados.

A normalidade da distribuição das variáveis contínuas foi avaliada pelo teste de Kolmogorov-Smirnov e a homogeneidade da distribuição entre os grupos pelo Teste de Levene. Como foi observado na distribuição não normal para todos os escores clínicos, estes estão descritos através de mediana e do intervalo interquartil. A comparação de suas distribuições entre os grupos foi confirmada pelo Teste de Mann-Whitney, não paramétrico.

Não houve o cálculo do tamanho amostral, sendo incluídos no estudo todos os pacientes que atenderam aos critérios de inclusão e que concordaram em participar. A associação entre o escore SYNTAX e os demais escores de risco, TIMI, GRACE e HEART, foi avaliada a partir de correlações bivariadas e através da adoção do coeficiente de Spearman para variáveis não paramétricas.

Para que se investigasse a acurácia dos escores TIMI, GRACE e HEART em identificar pacientes com escore SYNTAX moderado e alto, foram utilizadas as curvas ROC. Duas variáveis binárias foram criadas, classificando grupos de pacientes com SYNTAX baixo *versus* moderado-alto (≥ 23) e baixo-moderado versus alto (> 32). Os cálculos de sensibilidade e especificidade foram realizados com base nos pontos de corte previamente descritos dos escores TIMI, GRACE e HEART. A significância estatística foi estabelecida em 5% (p < 0,05). O *software* estatístico utilizado na análise dos dados foi o SPSS, versão 25.

## Resultados

No período de julho de 2018 a fevereiro de 2019, foram internados 292 pacientes com SCA, dos quais 105 (35,9%) foram excluídos por não terem sido submetidos ao cateterismo cardíaco, 24 (8,2%) tinham o diagnóstico de IAMCSST e 25 (8,6%) apresentavam histórico de RM. Todos os pacientes elegíveis foram analisados de forma sequencial.

A Tabela 1 apresenta a caracterização da amostra final. Dos 138 pacientes analisados, 68,1% eram do sexo masculino, a média de idade foi de 60±13 anos, e 32,2% apresentaram IAMSST (Tabela 1). As medianas (intervalo-interquartil) do GRACE, TIMI e HEART foram de 96,5 (76,5-115,7), 2,8 (2-4) e 5,0 (4-6), respectivamente. Não foi observada estenose coronária significativa em 29,7% dos pacientes, enquanto 43,7% dos pacientes apresentaram doença multiarterial. Os três escores clínicos foram significativamente mais altos nos pacientes com SYNTAX moderado ou alto, quando comparados com o grupo de pacientes com SYNTAX baixo (Tabela 2).

**Tabela 1:** Características da população estudada

	Total	SYNTAX < 23	SYNTAX ≥ 23	p	SYNTAX ≤ 32	SYNTAX > 32	p
N (%)	138 (100)	114 (82,6)	23 (16,7)		126 (91,3)	11 (8,0)	
Sexo masculino, n (%)	94 (68,1)	77 (73,9)	17 (73,9)	0,63	86 (68,3)	8 (72,7)	1,0
Idade, média ± dp	60,2 ± 11,3	59,4 ± 10,7	65,0 ± 9,2	0,02	59,4 ± 10,9	67,4 ± 10,6	0,02
IMC, média ± dp	27,9 ± 4,9	27,9 ± 5,1	27,9 ± 3,9	0,94	27,8 ± 4,9	28,9 ± 4,9	0,49
Obesidade, n (%)	39 (28,3)	34 (30,6)	5 (21,7)	0,46	37 (30,1)	2 (18,2)	0,51
Diabetes, n (%)	50 (36,2)	41 (36,0)	9 (39,1)	0,82	44 (34,9)	6 (54,5)	0,21
Dislipidemia, n (%)	72 (52,2)	62 (54,4)	10 (45,5)	0,49	68 (54,4)	4 (36,4)	0,35
Hipertensão, n (%)	115 (83,3)	94 (82,5)	20 (87,0)	0,76	105 (83,3)	9 (81,8)	1,0
Tabagismo, n (%)	37 (26,8)	33 (28,9)	4 (17,4)	0,31	35 (27,8)	2 (18,2)	0,73
Sedentarismo, n (%)	132 (95,7)	109 (96,5)	22 (95,7)	1,0	120 (96,0)	11 (100)	1,0
Cr, mediana (IIQ)	0,9 (0,7-1,0)	0,9 (0,7-1,0)	0,8 (0,7-1,1)	0,89	0,9 (0,7-1,0)	0,8 (0,7-1,1)	0,97
**Diagnóstico, n (%)**							
Angina instável	93 (67,3)	80 (70,2)	13 (56,5)	0,23	89 (70,6)	4 (36,6)	0,04
IAMSSST	45 (32,6)	34 (29,8)	10 (43,5)		37 (29,4)	7 (45,5)	

Estatística: Teste de Qui-quadrado para a comparação de proporções e modelos lineares generalizados (GLM) para comparação das variáveis contínuas. DP: desvio-padrão; IMC: índice de massa corpórea; Cr: creatinina; IIQ: intervalo interquartil; IAMSSST: infarto agudo do miocárdio sem supradesnivelamento do segmento ST. Define-se obesidade como IMC > 30 kg/m^2^.

**Tabela 2:** Diagnóstico e desfechos hospitalares

	Total	SYNTAX < 23	SYNTAX ≥ 23	p	SYNTAX ≤ 32	SYNTAX > 32	p
N (%)	138 (100)	114 (82,6)	23 (16,7)		126 (91,3)	11 (8)	
**Dias internação**							
Mediana (IIQ)	3 (2-6)	3 (2-5)	8 (3-20)	< 0,001	3 (2-5)	14 (7-23)	< 0,001
**Via acesso, n (%)**							
Radial	97 (70,3)	82 (71,9)	14 (60,9)	0,32	90 (71,4)	6 (54,5)	0,30
Femoral	41 (29,7)	32 (28,1)	9 (39,1)		36 (28,6)	5 (45,5)	
**N de vasos, n (%)**							
Sem DAC	41 (29,7)	41 (36,0)	0		41 (32,5)	0	
Uniarterial	42 (30,4)	42 (36,8)	0	< 0,001	42 (33,3)	0	< 0,001
Biarterial	20 (14,6)	18 (15,8)	2 (8,7)		18 (14,3)	2 (18,2)	
Triarteraial	34 (39,1)	13 (11,4)	21 (91,3)		25 (19,9)	9 (81,8)	
**TCE, n (%)**	13 (9,4)	7 (6,1)	6 (26,1)	0,001	8 (6,3)	5 (45,5)	0,001
**GRACE**							
Mediana (IIQ)	97 (77-115)	93 (75-112)	105 (92-140)	0,020	94 (75-112)	140 (103-175)	< 0,001
> 139, n (%)	9 (6,5)	2 (1,8)	7 (31,8)	< 0,001	3 (2,4)	6 (54,5)	< 0,001
ASC			0,66 (0,53-0,79)		0,76 (0,53-0,79)		
**TIMI**							
Mediana (IIQ)	3 (2-4)	3 (2-3)	3 (2-5)	0,024	3 (2-3)	5 (3-6)	0,004
≥ 5. n (%)	16 (11,6)	9 (7,9)	7 (30,4)	0,006	10 (54,4)	6 (54,5)	< 0,001
ASC		0,66 (0,53-0,79)			0,81 (0,64-0,97)		
**HEART**							
Mediana (IIQ)	5 (4-6)	4 (4-6)	6 (5-8)	< 0,001	5 (4-6)	7 (5-8)	0,001
≥ 7 n (%)	26 (18,8)	18 (15,8)	8 (34,8)	0,044	20 (15,9)	6 (54,5)	0,006
ASC		0,72 (0,62-0,83)			0,81 (0,70-0,92)		
**SYNTAX**							
Mediana (IIQ)	8 (0-17)	6 (0-12)	32 (26-34)	< 0,001	7 (0-14)	34 (33-35)	0,001

Estatística: Teste de Qui-quadrado para a comparação de proporções e modelos lineares generalizados (GLM) para a comparação das variáveis contínuas. IIQ: intervalo interquartil; ASC: área sob a curva ROC; DAC: doença arterial coronária; TCE: tronco de coronária esquerda.

**Figura 1:**
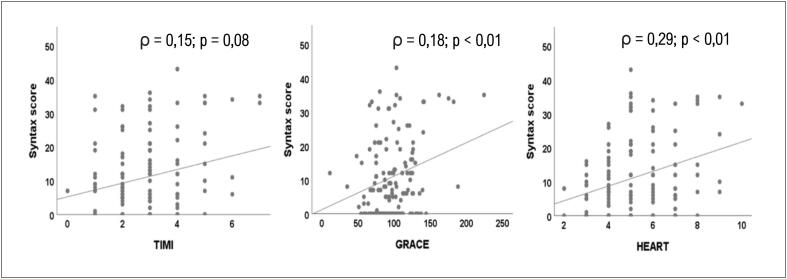
Gráficos de dispersão entre os valores numéricos dos escores do STYNTAX vs. TIMI, GRACE e HEART. Observação: (ρ) – Coeficiente rho de Spearman.

A Figura 1 é um gráfico de dispersão que apresenta a correlação entre os três escores clínicos e o SYNTAX. Foi observada correlação modesta em relação ao HEART (ρ = 0,29; p < 0,01) e ao GRACE (ρ = 0,18; p < 0,01), entretanto a correlação ao TIMI não atingiu significância estatística (ρ = 0,15; p = 0,08).

**Figura 2:**
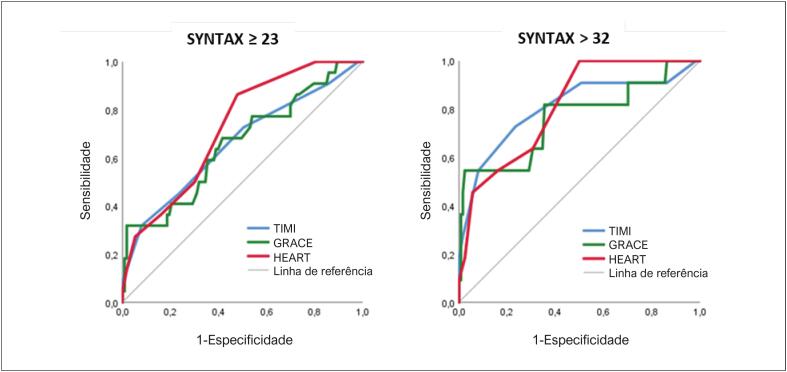
Curvas ROC para detecção de SYNTAX moderado ou elevado, de acordo com os escores de TIMI, GRACE e HEART. A – GRACE com ASC de 0,66 (IC 95% 0,53-0,79, p < 0,01); TIMI 0,66 (IC 95% 0,53-0,79); HEART 0,72 (IC 95% 0,62-0,83), p < 0,01. B – GRACE com ASC de 0,76 (IC 95% 0,53-0,79); TIMI 0,79 (IC 95% 0,64-0,97); HEART 0,81 (IC 95% 0,70-0,91), p < 0,01.

Na avaliação da curva ROC, ficou evidenciada acurácia similar entre os escores clínicos em predizer SYNTAX alto (> 32). A área sob a curva (ASC) ROC na associação ao HEART foi de 0,81 (IC 95% 0,7-0,91, p < 0,01), no TIMI de 0,79 (IC 95% 0,64-0,97), e no GRACE de 0,76 (IC 95% 0,53-0,79). (Figura 2).

O escore HEART maior que 5 apresentou sensibilidade de 64% e especificidade de 70% para avaliar SYNTAX alto (> 32). Quando maior que 4, apresentou sensibilidade de 100%, com especificidade de 50%.

O escore GRACE maior que 102 conferiu sensibilidade de 82% e especificidade de 65%. No ponto de corte original maior que 139 dá sensibilidade de 55%, mas apresenta especificidade de 97%. Dessa forma, com a utilização de escores GRACE e HEART de forma combinada, foi possível avaliar mais acuradamente para a predição de complexidade anatômica.

Durante a internação, observou-se um caso de óbito (0,72%) e outro de reinfarto (0,72%), sendo que os pacientes tinham SYNTAX de 33 e 19, respectivamente, e apresentavam os seguintes escores GRACE, TIMI e HEART: 69, 5 e 5 para primeiro; e 126, 1 e 5 para o segundo.

## Discussão

O presente estudo avaliou os três escores clínicos mais relevantes e utilizados no contexto de SCASSST. Os escores GRACE e TIMI já foram extensamente estudados e validados em diversas populações por serem capazes de predizerem eventos clínicos desfavoráveis e ambos são recomendados pelas principais diretrizes internacionais.^1^^,^^2^

O escore HEART vem sendo cada vez mais utilizado no contexto de dor torácica aguda na sala de emergência, devido ao alto valor preditivo negativo e a sua capacidade de evitar internações desnecessárias. Entretanto, valores elevados no escore HEART também possuem reconhecida capacidade de predizer eventos desfavoráveis, o que torna plausível avaliar a sua associação à complexidade anatômica.^6^^,^^13^

O reconhecimento da extensão da doença arterial coronária, através da identificação de fatores prognósticos clínicos, possui relevância notória na definição da melhor estratégia de revascularização e da terapia medicamentosa ideal. Dessa forma, foi utilizado o escore SYNTAX como forma de quantificar a extensão da doença coronariana.

Alguns estudos já avaliaram a associação do TIMI e do GRACE ao número de artérias acometidas. Mahmood et al. demonstraram que valores de TIMI maiores que 4 ou de GRACE maiores que 133 estão associados a maior probabilidade de doença multiarterial ou de estenose significativa em tronco de coronária esquerda (p < 0,05). Bakler et al. avaliaram a associação desses mesmos escores clínicos à complexidade anatômica através do SYNTAX, como no presente estudo, mas não incluindo o HEART. Assim como o presente estudo, foi evidenciada a associação linear positiva entre SYNTAX e GRACE, com um coeficiente de relação de r = 0,43 (p < 0,01) e ASC de 0,65 (IC 95% 0,56-0,74; p < 0,001). De forma semelhante, também não foi observada associação ao TIMI (r = 0,121, p = 0,121). Convém ressaltar que foram incluídos na amostra pacientes com IAMCSST (46% da amostra), contexto em que não é habitual a utilização do escore GRACE e não há validação para o cálculo do SYNTAX.^14^

Hammami et al.,^15^ avaliaram de forma retrospectiva o GRACE e o TIMI de 238 pacientes e observaram que ambos os escores apresentaram correlação positiva com SYNTAX. A associação ao GRACE apresentou relação de Pearson de r = 0,23 (p < 0,001) e com o TIMI de r = 0,2 (p = 0,002). Esses valores também foram comparáveis aos observados no presente estudo. Convém ressaltar que o estudo de Hammami et al. considerou, ao calcular o SYNTAX, lesões em mais de 70% dos casos. Apesar de plausível, tal forma de análise não apresenta validação por calculadoras oficiais ou estudos que mostrem sua acurácia ou seu prognóstico.^15^ Em estudo recente, Silvano et al.,^16^ avaliaram 183 pacientes com diagnóstico de IAMCSST (29,5%) e também observaram correlação modesta entre os escores de GRACE e SYNTAX (r = 0,2, p = 0,005). Os escores de TIMI e HEART não foram avaliados.^16^

Entre os estudos publicados que avaliam a associação entre os escores de risco e complexidade anatômica, observou-se, portanto, alguma correlação linear entre GRACE e SYNTAX, com resultados controversos quando utilizado o TIMI, similar ao resultado observado no presente estudo.

Esse é o primeiro estudo a fazer análise combinada dos escores GRACE e HEART para a associação à complexidade anatômica e que demonstrou aumento significativo da acurácia na predição de complexidade angiográfica quando os escores clínicos são utilizados simultaneamente.

Convém ressaltar que, no presente estudo, quando o escore HEART foi maior que 4, a sensibilidade foi de 100%, com especificidade de 50%; e quando GRACE maior que 139, a sensibilidade foi de 55%, com especificidade de 97% para SYNTAX alto. Esse estudo gera, portanto, a hipótese de que, em cenários específicos de SCASSST com escores de risco clínico elevados (GRACE > 139 e HEART > 4), a equipe e o paciente possam se preparar para uma possível abordagem cirúrgica, pela maior probabilidade de SYNTAX elevado.

No que diz respeito às limitações, convém mencionar o número pequeno de pacientes, em estudo realizado em centro único, bem como a ausência de um segundo avaliador para revisão do SYNTAX e demais escores. Devido ao baixo número de ocorrência e curto período de seguimento, não houve poder estatístico suficiente para que se investigasse a relação entre os escores clínicos e os desfechos como a mortalidade e o reinfarto.

## Conclusão

Os escores clínicos de risco apresentam associação modesta ao escore SYNTAX. O uso combinado do HEART e do GRACE, entretanto, oferece acurácia favorável para a detecção de complexidade angiográfica.
