# Supplementary material for: Associação entre Escores de Risco Clínico (HEART, GRACE e TIMI) e Complexidade Angiográfica na Síndrome Coronária Aguda sem Elevação do Segmento ST
Source: Arq Bras Cardiol. 2021 Aug 9;117(2):281–7. [Article in Portuguese] doi: 10.36660/abc.20190417 (PMC8395810; doi:10.36660/abc.20190417)
Supplement: Translation

# Translation: Association between Clinical Risk Score (Heart, Grace and TIMI) and Angiographic Complexity in Acute Coronary Syndrome without ST Segment Elevation

**Authors:** Alexandre Vianna Cedro, Diandro Marinho Mota, Louis Nakayama Ohe, Ari Timerman, José Ribamar Costa, Laura de Siqueira Castro

**Affiliations:** 1 São PauloSP Brazil Instituto Dante Pazzanese de Cardiologia, São Paulo, SP - Brazil; 2 São PauloSP Brazil Universidade Federal de São Paulo Escola Paulista de Medicina, São Paulo, SP - Brazil

**Keywords:** Acute Coronary Syndrome, Organ Dysfunction Scores, Hospitalization, Thrombosis, Myocardial Infarction, Angiography/complications

## Abstract

**Background:**

GRACE, TIMI and HEART scores have been previously validated to predict serious untoward events among patients with non-ST elevation acute coronary syndrome (Non-ST ACS). However, the ability of these scores to discriminate the angiographic complexity of coronary artery disease has not been clearly established.

**Objectives:**

We sought to evaluate the correlation between clinical scores (TIMI, GRACE and HEART) and the anatomical complexity assessed by SYNTAX score, among non-ST ACS patients undergoing cinecoronariography.

**Methods:**

Transversal cohort encompassing patients with diagnosis of Non-ST ACS referred to invasive stratification in our single center, between July 2018 and February 2019. Association between the scores was established by the Pearson's linear correlation test while the accuracy of the clinical scores versus SYNTAX score was determined with the ROC curve.

**Results:**

A total of 138 patients were enrolled. Median GRACE, TIMI and HEART scores were 97, 3 and 5, respectively, whereas the median SYNTAX was 8. There was a positive correlation between the SYNTAX and the HEART (ρ =0.29; p<0.01) and GRACE (ρ =0.18; p<0.01) scores, but the correlation with TIMI reached no statistical significance (ρ =0.15; p=0.08). The HEART score was also the one with the highest area under the curve to predict a SYNTAX ≥32 [HEART = 0.81 (IC95% 0.7-0.91). HEART> 4 presented 100% sensitivity, with 50% specificity; and GRACE> 139 showed 55% sensitivity and 97% specificity for high SYNTAX.

**Conclusion:**

The clinical scores presented a positive, although modest, association with the SYNTAX score. The combined use of HEART and GRACE offers good accuracy for detecting angiographic complexity.

## Introduction

Non-ST elevation acute coronary syndrome (Non-ST ACS) has a broad spectrum of severity, which varies according to electrocardiographic, clinical, and laboratory characteristics. Thus, risk stratification is fundamental in every patient with Non-ST ACS and directly influences initial management. It has been shown that using multivariate models is the most accurate way to predict risk, being superior to clinical impression alone.^1^^,^^2^

The Thrombolysis in Myocardial Infarction (TIMI), Global Registry for Acute Coronary Events (GRACE), and Heart Score (HEART) are the most commonly used scores in patients with chest pain in the emergency room and have been validated to predict undesirable clinical outcomes. However, these scores are not intended to estimate the extent of coronary artery disease.^3^^–^^6^

The evaluation of anatomical complexity using the SYNTAX score is as fundamental in the definition of revascularization strategy as the analysis of clinical scores, with important prognostic implications.^7^

The SYNTAX study, which gave rise to the score, compared late clinical outcomes in patients requiring multiple grafts treated with angioplasty (PCI) and coronary artery bypass grafting (CABG). The authors found that CABG had a more favorable outcome than PCI in patients with more extensive coronary artery diseases (SYNTAX ≥ 33).^8^ Thus, the determination of the SYNTAX score may also conflict with the clinical approach, supporting the decision to use dual antiplatelet therapy when the anatomy is favorable for the surgical approach.^7^^,^^8^

Despite the importance of identifying prognostic factors dependent on the extent of coronary disease, few studies have investigated the correlation between clinical scores and anatomical complexity. Controversial results have been reported when the TIMI and GRACE scores have been used to analyze this relationship, and no study has associated the HEART score with the complexity of coronary artery disease.^9^^–^^11^

Therefore, this study aims to evaluate the association between TIMI, GRACE, and HEART^12^ scores and the complexity of coronary artery disease revealed by coronary angiography in patients with Non-ST ACS.

## Methods

### Population selection

This is an observational and longitudinal study conducted at the Dante Pazzanese Institute of Cardiology (São Paulo-SP, Brazil) between July 2018 and February 2019 and approved by the research ethics committee of the institution. All patients signed an informed consent form at the time of hospitalization.

We included patients over 18 years old admitted with a diagnosis of non-ST ACS in the emergency department and who underwent coronary angiography during hospitalization. Patients with CABG, ST-segment elevation acute myocardial infarction (STEMI), or presumed new left bundle-branch block were excluded.

### Clinical scores

All patients were evaluated and stratified by the HEART, TIMI, and GRACE scores at the time of hospitalization. Clinical data, admission electrocardiogram, the first creatinine dose, and the highest troponin value in the first 12 hours of care were used to calculate the scores. Patients were considered as having a high troponin level when their ultrasensitive troponin T value was ≥0.01 µg/L, that is, higher than the 99th percentile of the general population.

Criteria previously defined in validation studies were used to calculate the scores. TIMI score was calculated through the seven usual dichotomous variables. The presence of each variable added one point to the total score, which ranged from zero to seven. A TIMI score ranging from 5 to 7 was considered high.^3^

The GRACE score was calculated using eight variables and was revised using the score calculator (http://www.grace.org). A final score higher than 139 was considered high, as recommended in the main guidelines.^1^^,^^2^

The HEART score ranged from 0 to 10 according to its five usual variables (history, ECG, age, risk factors, and troponin value). After the calculation, patients with scores between 7 and 10 were classified as high risk.^5^

After conducting invasive risk stratification, all catheterizations were analyzed by an experienced interventional cardiologist. All analysis was performed by a single blind investigator, using Quantitative Coronary Analysis (QCA). The SYNTAX score was calculated and revised using the official score calculator (http://www.syntaxscore.com), checking the instructions and using the software available on the page.

Arteries with diameter ≥1.5 mm and stenosis ≥50% were evaluated. The score was defined for each patient according to the following parameters: dominance; number of lesions; presence of chronic occlusion, trifurcation, bifurcation, ostial lesion, severe tortuosity, calcification, and thrombus; and lesion length >20 mm. A narrowing of 50% of the lumen occurring 3 mm from the carina in an artery with branches of at least 1.5 mm indicated bifurcation. A radiopaque lesion observed even before the contrast injection indicated severe coronary calcification. After calculating the score, each patient was classified as having low- (≤ 22), moderate- (23-32) or high (≥ 33) SYNTAX score.^7^

### Statistical analysis

Data are represented as absolute frequency with percentage for categorical variables and as mean ± standard deviation (SD) or median with interquartile interval for continuous variables for continuous variables, according to normality and distribution criteria. Cross tables and the chi-squared test were used to compare proportions between groups, and analysis of variance was used to compare means. To compare the distribution of continuous variables we used General Linear Models

The normality of distribution of continuous variables was assessed by the Kolmogorov-Smirnov test, the homogeneity of distribution between groups by the Levene test. The results of the comparison between groups were confirmed by the nonparametric Mann-Whitney test.

Sample size was not calculated, with the number of patients being determined by simple scrutiny. The association between the SYNTAX score and other risk scores—TIMI, GRACE, and HEART—was evaluated using bivariate correlations adopting the Spearman coefficient for nonparametric variables.

ROC curves were used to determine whether the TIMI, GRACE, and HEART scores could accurately identify patients with moderate- and high SYNTAX scores. Two binary variables were created, stratifying patients by low versus moderate-high SYNTAX score (≥23) and low-moderate versus high SYNTAX score (>32). Sensitivity and specificity were calculated based on the previously described cutoff points of the TIMI, GRACE, and HEART scores. P values < 0.05 indicated statistical significance. The SPSS version 25 statistical software was used for data analysis.

## Results

From July 2018 to February 2019, 292 patients admitted with ACS and eligible for inclusion in the study were admitted. Of these, 105 (35.9%) individuals who did not undergo cardiac catheterization at that time, 24 (8.2%) who were diagnosed with STEMI, and 25 (8.6%) who had a history of CABG were excluded.

Table 1 shows the characteristics of the final sample. Of the 138 patients analyzed, 68.1% were male with a mean age of 60 ± 13 years, and 32.2% had NSTEMI (Table 1). The median of GRACE, TIMI, and HEART scores were 98.1 (76.5-115.7), 2.8 (2-4) e 5 (4-6), respectively. Significant coronary stenosis was not observed in 29.7% of patients, while 43.7% of patients required multiple grafts. All three clinical scores were higher in patients with moderate or high SYNTAX score than in those with low SYNTAX score (Table 2).

**Table 1:** Characterístics of the Participants at Baseline

	Total	Syntax <23	Syntax ≥23	p	Syntax ≤32	Syntax >32	p
N (%)	138 (100)	114 (82.6)	23 (16.7)		126 (91.3)	11 (8.0)	
Male, n (%)	94 (68.1)	77 (73.9)	17 (73.9)	0.63	86 (68.3)	8 (72.7)	1.0
Age, mean ± SD	60.2 ± 11.3	59.4±10.7	65.0±9.2	0.02	59.4±10.9	67.4±10.6	0.02
BMI, mean ± SD	27.9 ± 4.9	27.9 ± 5.1	27.9 ± 3.9	0.94	27.8 ± 4.9	28.9 ± 4.9	0.49
Obesity, n (%)	39 (28.3)	34 (30.6)	5 (21.7)	0.46	37 (30.1)	2 (18.2)	0.51
**Diabetes, n (%)**	50 (36.2)	41 (36.0)	9 (39.1)	0.82	44 (34.9)	6 (54.5)	0.21
Dyslipidemia, n (%)	72 (52.2)	62 (54.4)	10 (45.5)	0.49	68 (54.4)	4 (36.4)	0.35
Hypertension, n (%)	115 (83.3)	94 (82.5)	20 (87.0)	0.76	105 (83.3)	9 (81.8)	1.0
Smoking, n (%)	37 (26.8)	33 (28.9)	4 (17.4)	0.31	35 (27.8)	2 (18.2)	0.73
Sedentarism, n (%)	132 (95.7)	109 (96.5)	22 (95.7)	1.00	120 (96.0)	11 (100)	1.0
Cr, mediana (IIQ)	0.9 (0.7-1.0)	0.9(0.71-1.0)	0.8 (0.7-1.1)	0.89	0.9 (0.7-1.0)	0.8 (0.7-1.1)	0.97
Diagnosis, n(%)							
Unstable angina	93 (67.3)	80 (70.2)	13 (56.5)	0.23	89 (70.6)	4 (36.6)	0.04
NSTEMI	45(32.6)	34 (29.8)	10 (43.5)		37 (29.4)	7 (45.5)	

Statistics: Chi-square test for comparison of proportions and Generalized Linear Models (GLM) for comparison of continuous variables. BMI: body mass index; Cr: creatinine; NSTEMI: non-ST-segment acute myocardial infarction. Obesity was defined as BMI> 30 kg/m^2^

**Table 2:** Diagnosis and hospital outcomes

	Total	SYNTAX < 23	SYNTAX ≥ 23	p	SYNTAX ≤ 32	SYNTAX > 32	p
N (%)	138 (100)	114 (82.6)	23 (16.7)		126 (91.3)	11 (8)	
**Days of hosp. ± SD**							
Median (IIQ)	3 (2-6)	3 (2-5)	8 (3-20)	< 0.001	3 (2-5)	14 (7-23)	< 0.001
**Access, n (%)**							
Radial	97 (70.3)	82 (71.9)	14 (60.9)	0.32	90 (71.4)	6 (54.5)	0.30
Femoral	41 (29.7)	32 (28.1)	9 (39.1)		36 (28.6)	5 (45.5)	
**Access, n (%)**							
Without CAD	41 (29.7)	41 (36.0)	0		41 (32.5)	0	
One artery	42 (30.4)	42 (36.8)	0	< 0.001	42 (33.3)	0	< 0.001
Two arteries	20 (14.6)	18 (15.8)	2 (8.7)		18 (14.3)	2 (18.2)	
Three arteries	34 (39.1)	13 (11.4)	21 (91.3)		25 (19.9)	9 (81.8)	
**LMCA, n (%)**	13 (9.4)	7 (6.1)	6 (26.1)	0.001	8 (6.3)	5 (45.5)	0.001
**GRACE**							
Median (IIQ)	97 (77-115)	93 (75-112)	105 (92-140)	0.020	94 (75-112)	140 (103-175)	< 0.001
>139, n(%)	9 (6.5)	2 (1.8)	7 (31.8)	< 0.001	3 (2.4)	6 (54.5)	< 0.001
ASC		0.66 (0.53-0.79)			0.76 (0.53-0.79)		
**TIMI**							
Median (IIQ)	3 (2-4)	3 (2-3)	3 (2-5)	0.024	3 (2-3)	5 (3-6)	0.004
≥5. n(%)	16 (11.6)	9 (7.9)	7 (30.4)	0.006	10 (54.4)	6 (54.5)	< 0.001
ASC		0.66 (0.53-0.79)			0.81 (0.64-0.97)		
**HEART**							
Median (IIQ)	5 (4-6)	4 (4-6)	6 (5-8)	< 0.001	5 (4-6)	7 (5-8)	0.001
≥7 n (%)	26 (18.8)	18 (15.8)	8 (34.8)	0.044	20 (15.9)	6 (54.5)	0.006
ASC		0.72 (0.62-0.83)			0.81 (0.70-0.92)		
**SYNTAX**							
Median (IIQ)	8 (0-17)	6 (0-12)	32 (26-34)	< 0.001	7 (0-14)	34 (33-35)	0.001

Statistics: Chi-square test for comparison of proportions and Generalized Linear Models (GLM) for comparison of continuous variables. ASC: Area Under the ROC Curve; LMCA: Left Main Coronary Artery.

Figure 1 illustrates the correlation between the three clinical scores and SYNTAX. A modest correlation was observed in relation to HEART (ρ = 0.29; p <0.01) and GRACE (ρ = 0.18; p <0.01), however the correlation with TIMI did not reach statistical significance (ρ = 0.15; p = 0.08).

**Figure 1:** Scatter plots between the numerical values of the SYNTAX scores vs. TIMI, GRACE, and HEART scores. Note: (ρ) Spearman's rho coefficient.

When evaluating the ROC curve, we observed that raised levels of all clinical scores could accurately predict a high SYNTAX score (>32). The association of SYNTAX score with HEART, TIMI, and GRACE scores resulted in an area under the receiver operating characteristic ROC curve (AUC) of 0.81 (95% CI 0.7-0.91, p < 0.01), 0.79 (95% CI 0.64-0.97), and 0.76 (95% CI 0.53-0.79), respectively. (Figure 2)

**Figure 2:** ROC curves for detecting moderate or high SYNTAX scores, according to TIMI, GRACE, and HEART scores. A – GRACE score with AUC of 0.66 (95% CI 0.53-0.79, p < 0.01); TIMI score of 0.66 (95% CI 0.53-0.79); HEART score of 0.72 (95% CI 0.62-0.83), p < 0.01. B – GRACE score with AUC of 0.76 (95% CI 0.53-0.79); TIMI score of 0.79 (95% CI 0.64-0.97); HEART score of 0.81 (95% CI 0.70-0.91), p < 0.01.

HEART greater than 5 showed 64% sensitivity and 70% specificity to evaluate high SYNTAX (>32). When greater than 4, it presented 100% sensitivity, with 50% specificity.

GRACE greater than 102 gave 82% sensitivity and 65% specificity. At the original cutoff point> 139 gives 55% sensitivity but 97% specificity. Thus, using both scores (GRACE and HEART), a more accurate assessment was possible to predict anatomical complexity.

Due to the low number of endpoints and a short follow-up period, there was not enough statistical power to investigate the relationship between clinical scores and outcomes such as mortality and reinfarction. There was 1 case of death (0.72%) and 1 case of reinfarction (0.72%), with patients having SYNTAX of 33 and 19, respectively, and the GRACE, TIMI and HEART scores of 69, 5 and 5 in the first and 126, 1 and 5 in the second.

## Discussion

The present study evaluated the three most relevant clinical scores used in the context of Non-ST ACS. The GRACE and TIMI scores have been extensively studied and validated in several populations due to their ability to predict unfavorable clinical events, being recommended by the main international guidelines.^1^^,^^2^

The HEART score has been increasingly used in patients with acute chest pain in the emergency room due to its high negative predictive value and ability to reduce unnecessary hospitalizations. However, high values of HEART score are known to predict unfavorable events so that it is worth investigating whether HEART score is associated with anatomical complexity.^6^^,^^13^

The determination of the extent of coronary artery disease, through the identification of clinical prognostic factors, plays an important role in the definition of the best revascularization strategy and ideal drug therapy. Thus, the SYNTAX score was used to quantify the extent of coronary disease.

Some studies have already evaluated the association between TIMI and GRACE scores with the number of affected arteries. Mahmood et al. found that TIMI > 4 or GRACE > 133 are associated with a higher probability of the patient requiring multiple grafts or having significant stenosis in the left main coronary artery (p < 0.05). Bakler et al. evaluated the association of clinical score with anatomical complexity using the SYNTAX score. A positive linear association between SYNTAX score and GRACE score was observed, with a ratio coefficient of r = 0.43 (p < 0.01) and AUC of 0.65 (CI 95% 0.56-0.74; p < 0.001). TIMI score was not associated with SYNTAX score (r = 0.121, p = 0.121), and HEART score was not evaluated. It should be noted that patients with STEMI (46% of the sample) were included, and these patients are not usually evaluated using the GRACE score.^14^

Hammami et al.^15^ retrospectively evaluated the GRACE and TIMI scores of 238 patients and observed that both scores showed positive correlation with the SYNTAX score. A Pearson correlation coefficient of r = 0.23 (p < 0.001) was found between SYNTAX score and GRACE score and a Pearson correlation coefficient of r = 0.2 (p = 0.002) was found between the SYNTAX score and the TIMI score. These values were similar to those observed in Figure 1, which showed a slightly higher correlation when comparing the GRACE (r = 0.26; p < 0.01) and the TIMI (r = 0.24; p < 0.01) scores. It should be noted that we only considered lesions > 70% when calculating the SYNTAX score. Although plausible, this form of analysis is not validated by official calculators or studies that investigate the accuracy of the score or the prognosis of patients.^15^ In a recent study, Silvano et al.^16^ evaluated 183 patients, including patients with STEMI (29.5%), and observed a positive but low correlation between GRACE and SYNTAX scores (r = 0.2, p = 0.005). TIMI and HEART scores were not evaluated.^16^

Some of the studies evaluating the association between risk scores and anatomical complexity reported a linear correlation between GRACE and SYNTAX scores, with controversial results when TIMI score was used, similar to the result observed in the present study.

This is the first study to do combined analysis of GRACE and HEART, demonstrating the significant increase in accuracy in predicting angiographic complexity when using both clinical scores simultaneously.

In this study, when the HEART score was greater than 4, the sensitivity was 100%, with a specificity of 50%; and when GRACE greater than 139 the sensitivity is 55% and specificity is 97% for high SYNTAX. Therefore, this study hypothesizes that, in specific scenarios of high clinical risk scores (GRACE> 139 and HEART> 4), the team and the patient can prepare for a surgical approach, due to the higher probability of high SYNTAX, by elevated SYNTAX.

One of the noteworthy limitations of this study is the small number of patients for a study conducted in a single center, as well as the absence of a second evaluator to analyze the methods and the other scores.

## Conclusion

The clinical scores presented a positive, although modest, association with the SYNTAX score. The combined use of HEART and GRACE, offers good accuracy for detecting angiographic complexity.
